# Pancreatic Metastases from Cervical Squamous Cell Carcinoma: Systematic Review of the Literature and Case Report

**DOI:** 10.3390/biomedicines13112713

**Published:** 2025-11-05

**Authors:** Siyuan Qian-Zhang, Diego Romero-Triana, Cecilia Meliga, Víctor Domínguez-Prieto, Begoña López-Botet Zulueta, Mario Martín-Sánchez, Santos Jiménez-Galanes, Enrique Rojo-Villardón, Pedro Villarejo-Campos

**Affiliations:** 1 Department of Surgery, Fundación Jiménez Díaz University Hospital, Avenida de los Reyes Católicos 2, 28040 Madrid, Spain; 2 Department of Radiology, Fundación Jiménez Díaz University Hospital, Avenida de los Reyes Católicos 2, 28040 Madrid, Spain; 3 Department of Surgery, Universidad Autónoma de Madrid, 28029 Madrid, Spain

**Keywords:** cervical cancer, pancreatic metastases, oncological surgery

## Abstract

**Background:** Pancreatic metastases from cervical cancer are exceptionally rare, with limited cases described in the literature. Their diagnosis and management remain challenging due to the absence of standardized protocols and the often poor prognosis. **Case Presentation:** We report the case of a 39-year-old woman with a history of treated stage IIIB cervical squamous cell carcinoma who presented with a solitary mass in the pancreatic tail. Diagnosis was established through cross-sectional imaging, PET-CT, and EUS-guided needle biopsy. The patient underwent systemic chemotherapy and SBRT followed by surgical resection. Histopathological analysis confirmed metastatic squamous cell carcinoma associated with HPV. Despite an initially favorable recovery, peritoneal metastases developed three months later. The patient died seven months after surgery under palliative care after disease progression on immunotherapy. **Methods:** A systematic review following PRISMA guidelines was conducted across PubMed, Cochrane, and Embase (2000–2025) to identify case reports and series describing pancreatic metastases from cervical cancer. A total of 14 published cases, together with the present case, were analyzed for demographic, clinical, diagnostic, therapeutic, and outcome data. **Results:** The mean age at diagnosis was 52.5 years (range 36–70). Squamous cell carcinoma was the predominant histology (73%). The pancreatic head was the most common metastatic site (53%). Diagnosis typically relied on abdominal CT, PET-CT, and EUS-guided biopsy. Surgical resection was performed in 28.6% of cases, while systemic therapy—most commonly cisplatin, paclitaxel, and bevacizumab—remained the mainstay for inoperable patients. **Conclusions:** Pancreatic metastases from cervical cancer usually occur in advanced disease stages and are associated with poor outcomes. Accurate diagnosis requires integration of imaging and histopathology, with PET-CT useful for detecting additional metastases. Surgery may be beneficial in selected patients with isolated lesions, but systemic therapy remains the primary treatment for most. Emerging immunotherapies show promise but are still in early development. Multidisciplinary management and further research are needed to optimize outcomes in this rare presentation.

## 1. Introduction

Cervical cancer (CC) is the fourth most common cancer among women, with approximately 660,000 new cases and 350,000 deaths reported in 2022. Over the past three decades, both its prevalence and mortality have risen substantially, particularly in Africa, where women aged 30–40 years are most affected [1,2]. Human papillomavirus (HPV) plays a central role in CC pathogenesis, with around 200 genotypes identified; high-risk subtypes 16 and 18 account for 70–80% of cases. Other risk factors include early onset of sexual activity, multiple sexual partners, and high-risk sexual behaviors. The natural history of the disease depends largely on the host immune response to primary HPV infection—leading to precursor lesions such as cervical intraepithelial neoplasia, spontaneous regression in approximately 90% of cases, or progression to malignancy [3,4].

The most frequent histological subtypes are squamous cell carcinoma (SCC) and adenocarcinoma (ADC), both arising in the “transformation zone,” with less common forms including adenosquamous carcinoma and neuroendocrine tumors. Once invasive disease develops, prognosis correlates closely with stage at diagnosis. For prognostic and therapeutic purposes, staging is performed using the AJCC/TNM and the International Federation of Gynecology and Obstetrics (FIGO) systems, classifying tumors as microinvasive or invasive (including recurrent and metastatic disease) [4,5].

Approximately 13% of patients are diagnosed at advanced stages, and around 2% present with distant metastases. Lymphatic metastasis refers to spread of lymph nodes beyond the pelvis (supraclavicular, mediastinal, para-aortic), while “hematogenous metastasis” involves solid organs, most often the lungs (36.3%), bones (16.3%), liver, brain, and other sites. Half of patients with hematogenous dissemination die within six months of diagnosis [6].

Metastatic CC carries a high mortality rate and poor response to available palliative therapies. Data on metastases to rare solid organs such as the pancreas remain scarce, both in diagnostic approaches and treatment strategies, highlighting the need to evaluate such presentations and report clinical experience in their management.

## 2. Case Report

A 39-year-old woman with a history of CC stage IIIB, diagnosed in February 2020 at another institution, previously treated with chemotherapy, radiotherapy, and brachytherapy (completed in May 2020), followed by right nephrectomy for ureteral obstruction. She remained under regular gynecological follow-up, showing complete radiological response and benign cytology findings, with no evidence of residual disease. She was only receiving anticoagulation therapy for deep venous thrombosis secondary to immobilization after a fracture.

**Figure 1:**
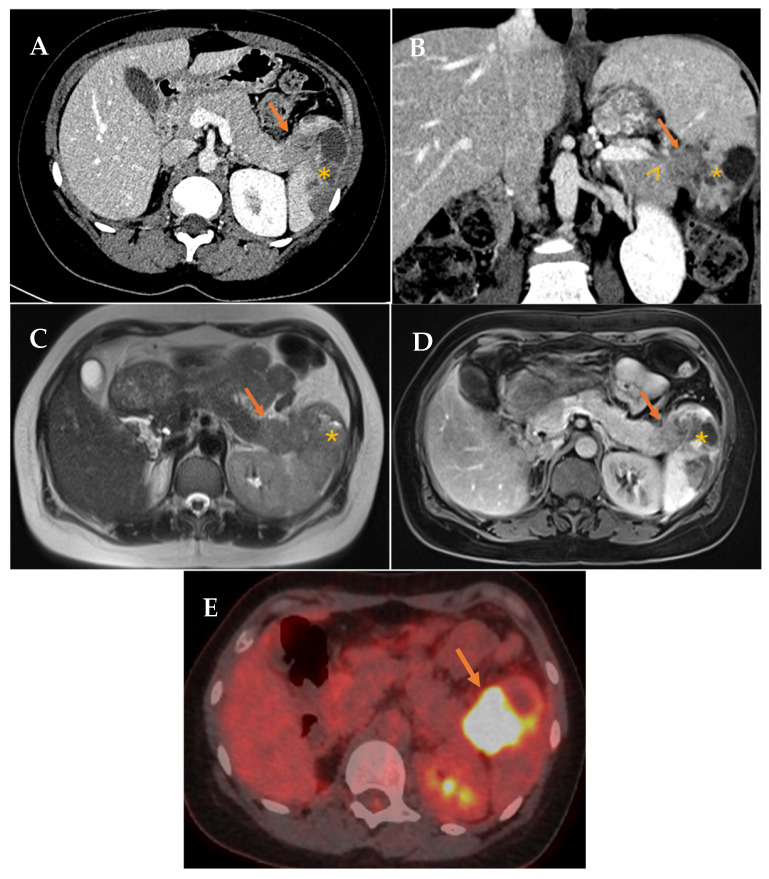
(**A**,**B**) Contrast-enhanced CT in portal phase. Axial and coronal reconstruction. Defined hypodense solid mass in the tail of the pancreas (arrow) with infiltration of the splenic hilum (arrowhead). Associated low attenuation changes in the spleen, secondary to infarction (asterisk). (**C**,**D**) Axial MRI. T2 and contrast-enhanced T1 sequences. Solid mass in the tail of the pancreas (arrow). Associated signal changes in the spleen, secondary to infarction (asterisk). (**E**) 18F-FDG PET/CT (18-fluorodeoxyglucose). Mass in the tail of the pancreas-splenic hilum region with pathological FDG uptake with splenic infiltration (arrow).

In July 2022, the patient reported left costal discomfort, prompting an Abdominal Computed Tomography (A-CT) that revealed an area of splenic infarction and a solitary mass measuring approximately 3 cm in the pancreatic tail/splenic hilum, suspicious for malignancy. Further workup with pancreatic Magnetic Resonance Imaging (MRI), 18F-fluorodeoxyglucose Positron Emission Tomography/CT (18F-FDG PET/CT) (Figure 1), and endoscopic ultrasound (EUS) with fine-needle aspiration (FNA) confirmed the diagnosis of squamous cell carcinoma, showing strong and diffuse positivity for CK 5/6 and p40.

She underwent systemic chemotherapy with paclitaxel, omitting cisplatin due to her single kidney, combined with stereotactic body radiation therapy (SBRT). Despite an initial favorable response after six cycles (completed in January 2023), follow-up PET-CT demonstrated a marked increase in lesion size and metabolic activity, although still confined to the pancreatic tail and splenic hilum, with no other pathological uptake or evidence of metastatic implants elsewhere.

After multidisciplinary discussion, surgical resection was recommended given the solitary nature of the lesion, its location in the pancreatic tail—which allowed a clear surgical approach—and the patient’s young age and good performance status. As preoperative imaging showed no signs suggestive of peritoneal or distant metastases, diagnostic laparoscopy was not proposed.

The patient underwent open distal pancreatectomy with splenectomy, gastric resection, and segmental colonic resection due (April 2023) to tumor infiltration identified intraoperatively; no evidence of peritoneal carcinomatosis or liver metastases was detected.

Histopathological examination revealed pancreatic and splenic parenchyma infiltrated by SCC, also involving the gastric and colonic walls. Three regional lymph nodes were identified, all negative for malignancy, and surgical margins were free of tumor (R0 resection). Histopathological analysis confirmed HPV-associated SCC (subtype 16), with p16 and p40 positivity on immunohistochemistry.

Three months postoperatively, imaging revealed development of the peritoneal carcinomatosis, confirmed by core biopsy. The patient was enrolled in a phase I clinical trial combining pembrolizumab with CLN-619, a humanized MICA/B-specific IgG1 monoclonal antibody. Unfortunately, the disease progressed, leading to gastric external compression and oral intolerance that required hospitalization. She was transitioned to palliative care and died seven months after surgery (November 2023).

**Figure 2:**
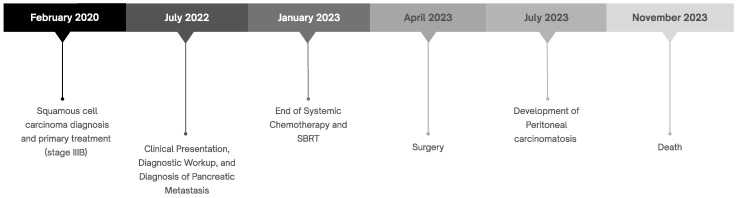
Overview of the patient’s clinical course summarized in a timeline.

A timeline summarizing the key milestones in the patient’s clinical course is provided to facilitate the understanding of the case evolution (Figure 2).

## 3. Materials and Methods

### 3.1. Systematic Review Design and Methodological Quality Assessment

A systematic review of the literature was conducted following the PRISMA (Preferred Reporting Items for Systematic Reviews and Meta-Analyses) 2020 guidelines, which provide a standardized framework to improve transparency, reproducibility, and methodological rigor in the identification, selection, and synthesis of studies. This review was registered in the OSF REGISTRIES (Open Science Framework) (https://osf.io/6enjr, accessed on 3 November 2025). The objective was to describe the general characteristics of the patients and tumors, identify diagnostic approaches, outline medical and surgical treatments, and report clinical outcomes.

Given the extreme rarity of the condition and the scarcity of scientific evidence, only descriptive studies, mainly individual case reports, were available for inclusion. A meta-analysis was not feasible due to the nature of the evidence (isolated case reports), marked heterogeneity in clinical presentation and management, small sample sizes, and the absence of comparison groups.

The systematic search was conducted between March and April 2025 in PubMed, Cochrane, and Embase. In PubMed, Medical Subject Headings (MeSH) were used, including “Pancreatic Neoplasms”, “Uterine Cervical Neoplasms”, and “Neoplasm Metastasis” combined with “Pancreas”. In the Cochrane Library, the search strategy included the terms “pancreatic neoplasm*”, “pancreatic mass*”, and “pancreas metastas*” combined with “uterine cervical neoplasm*”, “cervical cancer”, or “cervix carcinoma” using the Boolean operator AND. In Embase, the Emtree terms “pancreas tumor”, “uterine cervix cancer”, and “pancreas metastasis” were applied. These descriptors were combined using the Boolean operator AND (“Pancreatic Neoplasms” AND “Uterine Cervical Neoplasms”; “Uterine Cervical Neoplasms” AND “Neoplasm Metastasis”/“pancreas metastasis”), with the latter combination yielding the most relevant results. The complete search strategies for each database, including Boolean operators, MeSH/Emtree terms, and synonyms, are provided in Supplementary Materials, to ensure full reproducibility.

After the initial drafting of the manuscript, a second search was performed in October 2025 to ensure that no recent publications were missed, leading to the identification of one additional relevant article, which was incorporated into the review.

All citations were screened by two reviewers independently, and any discrepancies in study selection were resolved by a third reviewer. Similarly, all data were extracted by two reviewers and reviewed by a third reviewer to ensure accuracy and completeness. Articles published in any language were considered. Non-English studies were screened in their original language, and when selected for full-text review, they were translated using professional translation tools and verified by a bilingual reviewer to minimize language bias.

To assess the methodological quality of the included studies, the Joanna Briggs Institute (JBI) critical appraisal checklist for case reports was applied. This tool evaluates eight items per article, allowing classification of methodological quality as high (7–8 points), moderate (5–6 points), or low (<5 points). The completed PRISMA checklist and the JBI critical appraisal checklist are available in the Supplementary Materials.

### 3.2. Inclusion and Exclusion Criteria

1. Inclusion Criteria:
  - Keywords: *pancreatic neoplasm*, *cervical cancer*, *pancreatic metastases*
  - Year of publication: Articles published between 2000 and 2025.
  - Language: Any language.
  - Type of publication: case report, clinical case series and reviews.
2. Exclusion Criteria:
  - Pancreatic metastases with a primary tumor different from the cervix.

**Figure 3:**
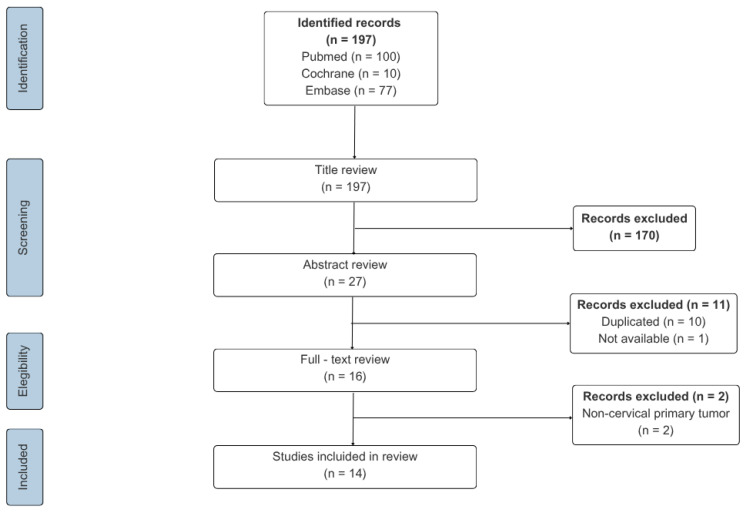
Flow chart of literature screening.

The article selection process is illustrated in Figure 3, and the key information from each report, including our own case, is summarized in Table 1.

## 4. Results

### 4.1. Search Results and Study Selection and Study Characteristics

The search retrieved 100 records in PubMed, 10 in Cochrane, and 77 in Embase. Based on the inclusion criteria, two reviewers independently screened all citations by title. 27 potentially relevant articles were identified. After removal of 10 duplicates and 1 inaccessible article, 16 full-text articles were reviewed, of which 2 were excluded because the primary tumor was not CC. Ultimately, 14 studies met the inclusion criteria and were analyzed together with the present case, yielding a total of 15 cases. All extracted data were reviewed by a third reviewer, who also resolved any discrepancies in study selection.

The selected publications reported pancreatic metastases (PM) originating from CC, providing a chronological description of the diagnostic process and, in most cases, the therapeutic approach. One case demonstrated secondary involvement of the ampulla of Vater; given its anatomical proximity and comparable diagnostic and therapeutic strategies, it was deemed appropriate to include it in the present series.

### 4.2. Patient and Tumor Characteristics

The mean age at diagnosis of PM was 52.5 years (range: 36–70), although in one case the exact age was not available; the author described the patient as middle-aged. The median age was 52.

Histologically, SCC was the most frequent subtype (11 cases, 73.3%), followed by ADC and small cell neuroendocrine tumor (SC-NET), with 2 cases each (13.3%).

According to the 2018 FIGO classification, Stage III was the most frequent at presentation (six cases, 40%), followed by Stage II (four cases, 26.7%), and Stages I and IV (one case each, 6.7%). Two cases (13.3%) had no staging data, and one (6.7%) initially presented as CIN3, later recurring as locally advanced squamous cell carcinoma without formal staging (Table 2).

### 4.3. Location of Pancreatic Metastases

The most frequent metastatic sites were the pancreatic head (eight cases, 53.3%) and body (five cases, 33.3%). Only our case involved the pancreatic tail (6.7%). One additional case involved the ampulla of Vater, included due to its anatomical proximity and comparable diagnostic and therapeutic approach (Table 3).

### 4.4. Diagnostic Tool

**Figure 4:**
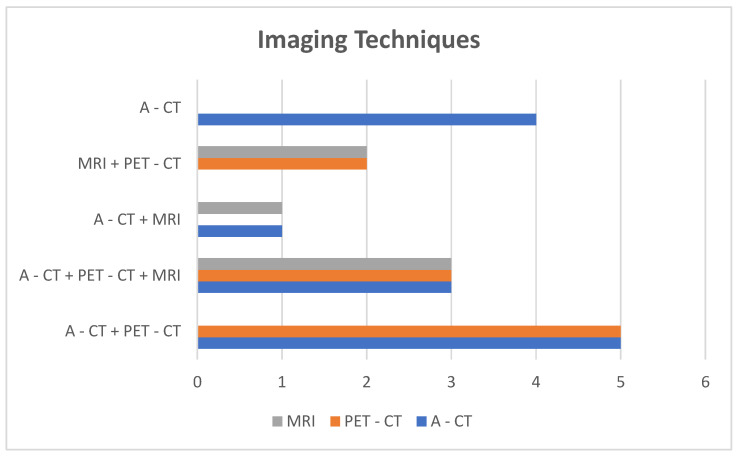
Imaging techniques and distribution groups.

- Imaging: A-CT was the initial diagnostic modality in thirteen patients, with MRI as the first-line test in two cases. As part of staging, nine patients underwent more than one imaging study: A-CT plus PET-CT (five cases), A-CT plus MRI (one case), and A-CT plus PET-CT plus MRI (three cases) (Figure 4).
- Invasive procedures: EUS was the most frequently used complementary diagnostic tool (ten cases). Other methods included EGD, ERCP, Guided biopsy-CT, and transendoscopic ultrasound-guided biopsy.
- No pre-treatment invasive procedures: Among the twelve patients treated medically or surgically, three (25%) received therapy without pre-treatment invasive procedures: one because of emergency surgery during diagnostic evaluation, one due to refusal of biopsy, and one with prior confirmation of metastasis by skin biopsy.

### 4.5. Medical Management

**Figure 5:**
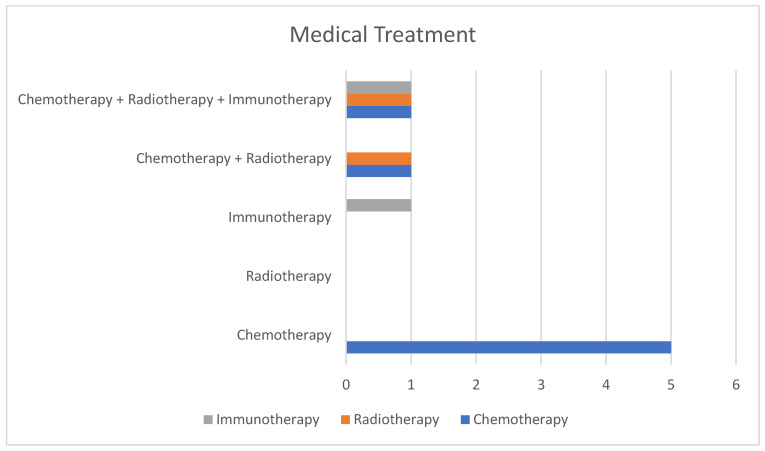
Imaging techniques and distribution groups.

- Non-surgical treatment for PM was reported in eight patients (53.3%) (Figure 5).
- Chemotherapy alone: five patients were treated exclusively with chemotherapy, using regimens that combined gemcitabine, cisplatin, etoposide, and paclitaxel.
- Radiotherapy alone: No patient was treated with radiotherapy alone.
- Immunotherapy alone: one case (pembrolizumab).
- Chemotherapy + radiotherapy: two cases.
- Chemotherapy + radiotherapy + immunotherapy: one case, initially treated with chemotherapy and immunotherapy, later adding radiotherapy for persistent disease, and finally maintained on immunotherapy (tislelizumab).

### 4.6. Surgical Management

Surgery for PM was performed in four patients (26.7%), including three elective cases and one emergency procedure.

- Emergency Surgery: Indicated for refractory gastrointestinal bleeding. The patient underwent exploratory laparotomy, subtotal pancreatectomy, splenectomy, total gastrectomy, left nephrectomy, partial colectomy, portal vein resection without reconstruction, and regional lymphadenectomy.
- Elective Surgery: among the three patients, one underwent surgery alone, one had surgery followed by adjuvant chemoradiotherapy, and one received neoadjuvant chemoradiotherapy, surgery, and adjuvant immunotherapy.

Regardless of the treatment modality received, medical or surgical, follow-up data across published cases were highly variable. Survival information was incomplete in several reports: in seven cases, patients were alive at the time of publication, and in four cases, survival time was not specified. Due to this heterogeneity and incomplete follow-up, it is difficult to establish a clear and reliable median survival for patients with pancreatic metastasis from cervical cancer.

## 5. Discussion

PM from CC is an exceptionally rare manifestation of the disease. Despite its low incidence, and to the best of our knowledge, this review represents the largest report to date in terms of number of patients with PM of cervical origin published in the scientific literature over the past two decades, encompassing a total of 14 cases. With the addition of the case managed by our team, the total rises to 15, enabling a detailed analysis of patient demographics, tumor characteristics, diagnostic strategies, and both medical and surgical management and outcomes.

CC is the fourth most common cancer and the third leading cause of cancer-related death among women. However, the increasing morbidity and mortality rates are predominantly observed in developing regions where approximately 90% of deaths occur. This is primarily attributed to the rising prevalence of HPV infection, limited access to early detection programs, and underdeveloped vaccination initiatives. An abnormal cytology or a positive HPV test typically leads to colposcopy and biopsy or excisional procedures, which may serve for diagnostic and therapeutic purposes. In its early stages, CC may be asymptomatic, whereas in locally advanced stages, it can present with abnormal uterine bleeding, vaginal discharge, dyspareunia, or chronic pelvic pain [21].

Histologically, CC is classified into three main groups: SCC (70–80%), ADC (20–25%), and other less common groups, including adenosquamous carcinoma and neuroendocrine tumor. Among these, SC-NET, which accounts for approximately 2% of cases, is characterized by its tendency for distant dissemination. Although definitive studies are lacking, ADC has traditionally been associated with poorer survival outcomes [1,21].

The clinical behavior of CC depends largely on its stage at diagnosis. According to the FIGO classification, locally advanced disease, regardless of histological subtype, corresponds to stages IB3 through IVA and is associated with a higher risk of locoregional spread and distant metastasis, and for which treatment response is variable. In our review, when all tumor types in the series are considered, including SCC, ADC, and SC-NET, the number of locally advanced cases increased to 11 (73.33%), consistent with previous reports indicating a higher risk of disease progression in such scenarios [22].

As the disease advances, CC typically spreads by direct extension to adjacent organs, most commonly affecting the bladder, vagina, and rectum [22]. Lymphatic dissemination to regional lymph nodes is also frequently observed. In contrast, distant hematogenous metastases are generally considered a late event in the disease course. Among distant metastatic sites, the lungs are the most commonly involved, followed by bone, liver, and, less frequently, the brain, which accounts for approximately 2% of cases. Notably, PM are exceptionally rare [5,23,24].

Prognosis varies significantly depending on the site of distant metastasis. Reported 5-year disease-specific survival rates progressively decline according to metastatic location: 14% for lung involvement, 10% for bone, 8% for liver, and 0% for brain metastases. Median survival for patients with hematogenous metastases is approximately 6 months [6,23].

Survival in patients with PM from CC remains poor, with no documented survival beyond 5 years, likely due to the extreme rarity of this presentation. In our review, survival outcomes varied widely across cases, reflecting differences in treatment modalities and follow-up duration. In some reports, survival was documented only up to the date of publication, which may underestimate the actual overall survival. This highlights both the aggressive nature of the disease and the limitations inherent to the available evidence, emphasizing the need for standardized long-term follow-up in future reports.

PM, in general, are rare, with a reported incidence ranging from 2 to 5%, and are typically associated with systemic dissemination. The most common primary tumor metastasizing to the pancreas is renal cell carcinoma. Other less frequent sources in Western populations include colorectal, breast, and lung cancers, as well as melanoma. Interestingly, in Eastern populations, particularly in China, lung, colorectal, and gastric cancers are reported as more frequent sources of PM [20,25].

Clinically, PM do not present with specific symptoms. As with primary pancreatic tumors, symptomatology depends on lesion location—obstructive jaundice when the tumor is located in the pancreatic head, while nonspecific symptoms such as abdominal discomfort, nausea, vomiting, or anorexia are more common when the tumor is located elsewhere in the pancreas, including the head [20,26]. Pancreatitis and gastrointestinal hemorrhage have also been documented [25]. Given the rarity of this condition, there are no large case series outlining a typical clinical course, nor are there well-established diagnostic or therapeutic algorithms.

The disparity in diagnostic methods or algorithms used for disease diagnosis and staging among groups reflects the lack of consensus. As Rampersad et al. specify, it is crucial to differentiate primary pancreatic neoplasia versus metastatic disease using methods that allow lesion identification (A-CT, CT-PET, MRI and EUS) and histopathological diagnosis [17]. Ye Hao et al. reinforce the importance of microscopic diagnosis, particularly in patients who may be surgical candidates. In this context, EUS-guided needle biopsy emerges as a highly valuable diagnostic tool [18].

Radiologically, PM may exhibit features suggestive of their extrapancreatic origin, including low attenuation, peripheral contrast enhancement, or distinctive vascular perfusion patterns, as seen on both contrast-enhanced CT and MRI. When hypervascular lesions are identified and multiple primary origins are possible, clinical suspicion, oncologic history, and histological confirmation via biopsy are essential for accurate diagnosis. While PET-CT has limited value in characterizing the pancreatic lesion itself, it plays a relevant role in detecting micrometastases throughout the body, thereby contributing to treatment planning and selection of the most appropriate therapeutic approach [25,27].

EUS-guided fine-needle biopsy allows cytological and histological confirmation of PM or recurrence, optimizing patient assessment and treatment selection. Its reported sensitivity and specificity are 93.8% and 60%, respectively, making it particularly valuable when imaging results are inconclusive. Although no pathognomonic radiological features have been established for PM, the presence of well-defined lesion margins appears to be a common characteristic [28,29].

In our systematic review, the mean age at diagnosis of PM was 53 years, which is notably higher than that observed in the patient treated by our team. This discrepancy may reflect the influence of additional risk factors contributing to the aggressiveness of the disease and its rapid progression. Among these, lack of HPV vaccination and delays in diagnosis and treatment may have negatively influenced both clinical presentation and therapeutic response [30].

From an anatomical standpoint, tumor location within the pancreas plays a critical role in management. In approximately 85% of the reported cases, the lesions were located in the head or body of the pancreas, regardless of patient age or initial disease stage. Nevertheless, the tumor’s anatomical location impacts the surgical strategy, thereby affecting morbidity and mortality outcomes [31].

In terms of therapeutic strategies, there is significant variability and debate regarding medical and surgical management. Some authors recommend surgery in potentially operable patients after confirmation of the metastatic origin. Conversely, in non-surgical candidates, individualized treatment based on the primary tumor and metastatic burden is considered appropriate. Akashi et al. reported that median survival following resection of PM from any origin does not exceed 31 months; results consistent with other series. Therefore, we emphasize the importance of individualized treatment planning and decision-making within multidisciplinary committees, given the wide range of medical and surgical options available [26].

Specifically, in cases of metastatic CC, international guidelines provide clear recommendations. The European Society for Medical Oncology (ESMO) guidelines recommend palliative chemotherapy as the standard approach for metastatic CC, primarily due to the typically symptomatic presentation and the overall poor prognosis. Similarly, the National Comprehensive Cancer Network (NCCN) also endorses chemotherapy as the first-line treatment. Both guidelines agree that the combination of cisplatin, paclitaxel, and bevacizumab constitutes the preferred first-line regimen. Additionally, immunotherapy with a monoclonal antibody targeting PD-1, in combination with standard chemotherapy, may also be considered as a first-line option. The addition of radiotherapy might also play an important role [21,32].

From a surgical perspective, suitable candidates for resection are operable patients with a well-controlled primary tumor of favorable prognosis and isolated PM, meeting resectability criteria similar to those for primary pancreatic neoplasms; such as the absence of major vascular invasion. The surgical approach depends on the location of the lesion and may include pancreaticoduodenectomy (with or without pyloric preservation), distal pancreatectomy, or total pancreatectomy. Some authors have proposed limited resections, such as enucleation or central pancreatectomy, aiming to reduce the risk of postoperative diabetes mellitus. However, these approaches have been less extensively studied and are associated with higher local recurrence rates, reported to be as high as 50%, as well as increased surgical morbidity. Therefore, achieving complete resection with negative margins and performing lymphadenectomy should remain the primary objectives of surgical management. The most important prognostic factor continues to be the type of primary tumor, with median postoperative survival reaching up to 30 months for renal cell carcinoma and approximately 26 months for other malignancies [25,31,33,34]. However, as mentioned earlier, PM are rare, and in the limited number of reported cases, median survival appears to be considerably shorter—approximately 7 months.

Palliative surgical or endoscopic interventions may be considered in patients who are not candidates for curative resection. Duodenal obstruction, which can lead to significant malnutrition, may be managed through surgical or endoscopic gastrojejunostomy. Similarly, in cases of biliary obstruction causing symptomatic jaundice, endoscopic placement of a biliary metal stent is an effective palliative measure [17,35].

Non-surgical treatment includes multiple strategies such as chemotherapy, radiotherapy, SBRT, and immunotherapy; the choice depends on the primary tumor and previous treatment response. All inoperable patients or those excluded from surgery will be evaluated by multidisciplinary teams to tailor treatment. Current guidelines recommend that cisplatin, paclitaxel, and bevacizumab constitute the preferred first-line regimen. The rise of immunotherapy (mainly anti-PD1/PD-L1 antibodies) within a multicomponent strategy has demonstrated benefit in PM. Additionally, radiotherapy in squamous cell carcinoma has shown higher remission rates although its use requires histological confirmation [20,21,32].

Recent advances in immunotherapy have enabled the development of Chimeric Antigen Receptor T (CAR-T) cells, which have shown remarkable efficacy in hematological malignancies and emerging potential in autoimmune diseases and solid tumors [36]. In CC, CART-T therapy remains at early stage, although preclinical studies demonstrate encouraging results. CAR-NK cells (Chimeric Antigen Receptor Natural Killer cell) targeting mesothelin have exhibited strong cytotoxicity against CC models [37], whereas the first clinical trial assessing mesothelin-directed CAR-T cells in solid tumors, including CC, was terminated early due to poor patient accrual, with one of 15 patients achieving stable disease [38]. The favorable safety profile and innate tumor-recognition ability of NK cells—attributed to absence of Major Histocompatibility Complex (MHC) molecule expression in NK and short lifespan—may underlie their potential as an alternative to CAR-T approaches [37,39]. Other strategies, including T-cell receptor-engineered T cells (TCR-T), are also under clinical evaluation in CC [40]. Ongoing research remains essential to define their therapeutic relevance and improve outcomes in refractory disease.

As a final consideration, the issue of fertility preservation deserves special attention, as it represents a crucial aspect of quality of life in young cervical cancer patients. Fertility preservation in this context remains a controversial and evolving topic, particularly relevant given that approximately 40% of women are diagnosed before the age of 45. The role of fertility-sparing treatments continues to be debated, especially in tumors larger than 2 cm. One of the main controversies concerns whether neoadjuvant chemotherapy should be administered prior to fertility-sparing surgery procedures—such as radical vaginal trachelectomy, abdominal trachelectomy, conization, or laparoscopic radical trachelectomy- or whether surgery should be performed upfront [41,42].

Finally, several limitations of this review must be acknowledged. These include the exclusive reliance on isolated case reports, which exhibit substantial heterogeneity in diagnostic assessments and therapeutic approaches, as well as incomplete data and follow-up in some publications, together with the retrospective nature of the available evidence. All included studies were individual case reports; therefore, no control group or comparative analysis of diagnostic or therapeutic strategies could be performed. A potential publication bias must also be considered, as unusual or favorable cases are more likely to be reported, potentially establishing the most effective management approach for patients with suspected pancreatic metastasis of cervical origin. Future studies should aim to minimize publication bias, ensure complete and standardized follow-up, and adopt uniform reporting criteria to improve evidence quality.

## 6. Conclusions

PM from CC are exceptionally rare and typically occur in locally advanced stages of the disease. Despite the limited number of cases reported, our review—comprising 15 patients including our own case—represents the most extensive review to date and highlights the challenges in diagnosis and management. Accurate identification through cross-sectional imaging and histopathological confirmation is essential, while PET-CT can assist in detecting micrometastases. Prognosis remains poor and no standardized treatment protocols exist. Surgical resection may be considered in highly selected cases, specifically in patients with a well-controlled primary tumor, isolated pancreatic metastasis, and favorable surgical criteria. For patients not amenable to surgery, systemic therapy remains the cornerstone of treatment. Current international guidelines recommend a first-line regimen combining cisplatin, paclitaxel, and bevacizumab, with anti-PD-1 immunotherapy now emerging as a valuable addition. Advances in adoptive immunotherapy, such as CAR-T and CAR-NK cells, offer potential but are still in early stages of clinical translation. Multidisciplinary decision-making and continued research will be key to improving outcomes in this rare and aggressive presentation.

## Figures and Tables

**Table 1:** Summary of identified case reports.

#	Author	Year	Age	Histology	HPV	Initial FIGO Stage	Imaging Techniques	Invasive Procedures	Localization	Other Site Metastases	Medical Treatment	Surgery	Survival *
1	Hoon Lee et al. [7]	2011	50	SCC	NR	IIA	A-CT, PET-CT	EGD, ERCP	Ampulla of Vater	No	Chemotherapy (NR)	No	NR
2	Ogawa et al. [8]	2011	45	SCC	NR	ND	A-CT	ERCP	Body	No	Radiotherapy (40 Gy/20 fr.), Chemotherapy (NR)	Emergency ^1^	Death 8 months after surgery
3	Ogandaga et al. [9]	2017	50	SCC	NR	CIN3	A-CT	Guided biopsy-CT	Body	No	No	No	Death 2 months after diagnosis without treatment
4	Mahajan et al. [10]	2017	57	ADC	NR	IIIA	A-CT, PET-CT	Transendoscopic ultrasound-guided biopsy	Head	No	Chemotherapy (Cisplatin, gemcitabine)	No	Alive at 4 months of follow-up
5	Kim et al. [11]	2018	70	ADC	+	IIB	A-CT, MRI, PET-CT	EUS, EUS-guided needle biopsy	Body	No	NR	NR	NR
6	Kopke et al. [12]	2018	56	SC-NET	NR	ND	A-CT, PET-CT	EUS, EUS-guided needle biopsy	Head	Liver, Retroperitoneum, Brain (during medical treatment)	Chemotherapy (1 line: cisplatin, etoposide) (2 line: paclitaxel)	No	Alive at 24 months of follow-up
7	Gupta et al. [13]	2019	55	SCC	NR	IIIB	A-CT	No	Body	Skin	No	Yes ^2^	Alive at 6 months of follow-up
8	Lee et al. [14]	2019	36	SC-NET	+	IB2	MRI, PET-CT	EUS, EUS-guided needle biopsy	Head Body	Breasts, Adrenal glands, Peritoneum	Chemotherapy (cisplatin, etoposide)	No	NR
9	Kudaravalli et al. [15]	2022	46	SCC	NR	IIB	A-CT	EUS, ERCP-guided needle biopsy	Head	No	Immunotherapy (pembrolizumab)	No	Alive in publication moment
10	Datta et al. [16]	2022	53	SCC	+	IIIC	A-CT, PET-CT	EUS, EUS-guided needle biopsy	Head	No	Chemotherapy (NR)	No	Alive at 6 months of follow-up
11	Rampersad et al. [17]	2022	51	SCC	NR	IVB	A-CT, PET-CT, EUS, ERCP, fine needly biopsy	EUS, ERCP-guided needle biopsy	Head	No	NR	NR	NR
12	YE Hao et al. [18]	2022	63	SCC	NR	IIIB	A-CT, MRI	EUS, ERCP	Head	No	Chemotherapy (NR)	Yes ^3^	Alive in publication moment
13	Nakajima et al. [19]	2023	ND	SCC	NR	IIB	A-CT, MRI, PET-CT	EUS, EUS-guided needle biopsy	Body	No	Radiotherapy (45 Gy/15 fr.), chemotherapy (docetaxel, carboplatin)	No	Death 7 months after diagnosis
14	Liao et al. [20]	2025	64	SCC	NR	IIIB	MRI, PET-CT	EUS, EUS-guided needle biopsy	Head	No	Radiotherapy (45 Gy/25 fr.), chemotherapy (paclitaxel, carboplatin), Immunotherapy (bevacizumab, tislelizumab)	No	Alive at 10 months of follow-up
15	Present Case	2023	39	SCC	+	IIIB	A-CT, MRI, PET-CT	EUS, EUS-guided needle biopsy	Tail	No	Chemotherapy (Paclitaxel), SBRT, Immunotherapy (pembrolizumab, CLN-619)	Yes ^4^	Death 7 months after surgery

Note: * Survival reported was in the publication moment. ^1^ Subtotal pancreatectomy, splenectomy, total gastrectomy, left nephrectomy, partial colectomy, portal vein resection without reconstruction, and regional lymph node dissection; ^2^ Distal pancreatectomy and splenectomy; ^3^ Pancreaticoduodenectomy; ^4^ Distal pancreatectomy and splenectomy, gastric resection, and segmental colonic resection. Abbreviations: ADC, adenocarcinoma; A-CT, abdominal computed tomography; CIN, Cervical Intraepithelial Neoplasia; EGD, esophagogastroduodenoscopy; ERCP, endoscopic retrograde cholangiopancreatography; EUS, endoscopic ultrasonography; MRI, Magnetic Resonance Imaging; NR, no results available in the article; PET-CT, positron emission tomography/computed tomography; SBRT, stereotactic body radiotherapy; SCC, squamous cell carcinoma; SC-NET, small cell neuroendocrine tumor.

**Table 2:** Stage of cervical cancer.

Stage	Total	
**Stage I**	**1**	6.7%
IA	0	
IB	1	
**Stage II**	**4**	26.7%
IIA	1	
IIB	3	
**Stage III**	**6**	40%
IIIA	1	
IIIB	3	
IIIC	1	
**Stage IV**	**1**	6.7%
IVA	0	
IVB	1	
**Other**	**3**	20%
CIN3	1	
No Data	2	

**Table 3:** Location of pancreatic metastases.

Location	Total	
**Head**	8	53% of the cases
**Body**	5	33% of the cases
**Tail**	1	7% of the cases
**Other**	1	7% of the cases

## Data Availability

All data generated or analyzed during this study are included in this published article and its Supplementary Materials. Additional information can be obtained from the corresponding author upon request.

## Supplementary Materials

The following supporting information can be downloaded at: https://www.mdpi.com/article/10.3390/biomedicines13112713/s1, the complete search strategies for each database, the completed PRISMA checklist and the JBI critical appraisal checklist.

## Abbreviations

The following abbreviations are used in this manuscript:

A-CT	Abdominal Computed Tomography
ADC	Adenocarcinoma
CAR-NK cell	Chimeric Antigen Receptor Natural Killer cell
CAR-T cell	Chimeric Antigen Receptor T cell
CC	Cervical cancer
EGD	Esophagogastroduodenoscopy
ERCP	Endoscopic Retrograde Cholangiopancreatography
EUS	Endoscopic Ultrasonography
FIGO	International Federation of Gynecology and Obstetrics
HPV	Human Papillomavirus
JBI	Joanna Briggs Institute
MHC	Major Histocompatibility Complex
MRI	Magnetic Resonance Imaging
NR	No Results
PET-CT	Positron Emission Tomography/Computed Tomography
PM	Pancreatic metastases
PRISMA	Preferred Reporting Items for Systematic Reviews and Meta-Analyses
SBRT	Stereotactic Body Radiotherapy
SC-NET	Small Cell Neuroendocrine Tumor
SCC	Squamous Cell Carcinoma
TCR-T	T-cell Receptor-engineered T cells
TIL	Tumor-Infiltrating Lymphocyte

## References

[1] Azangou-Khyavy M., Ghasemi E., Rezaei N., Khanali J., Kolahi A.-A., Malekpour M.-R., Heidari-Foroozan M., Nasserinejad M., Mohammadi E., Abbasi-Kangevari M. (2024). Global, regional, and national quality of care index of cervical and ovarian cancer: A systematic analysis for the global burden of disease study 1990–2019. BMC Women’s Health.

[2] Ferlay J., Colombet M., Soerjomataram I., Parkin D.M., Piñeros M., Znaor A., Bray F. (2021). Cancer statistics for the year 2020: An overview. Int. J. Cancer.

[3] Kobayashi O., Kamata S., Okuma Y., Nakajima T., Ikeda Y., Saito K., Kawana K. (2024). Carcinogenesis and epidemiology of cervical cancer: The hallmark of human papillomavirus-associated cancer. J. Obstet. Gynaecol. Res..

[4] Gil Moreno A., Coronado Martín P., Torné Bladé A., Saco Álvarez A., Velasco Alonso J., González Martín A., Corte J., Rosales A.L., Marti L., Sebastia J.P.I. (2018). Oncoguía SEGO: Cáncer de Cuello Uterino 2018. Oncoguía SEGO.

[5] Bhatla N., Aoki D., Sharma D.N., Sankaranarayanan R. (2021). Cancer of the cervix uteri: 2021 update. Int. J. Gynecol. Obstet..

[6] Li H., Wu X., Cheng X. (2016). Advances in diagnosis and treatment of metastatic cervical cancer. J. Gynecol. Oncol..

[7] Lee T.H., Park S.-H., Lee C.K., Lee S.-H., Chung I.-K., Kim S.-J., Kim S.W. (2011). Ampulla of Vater metastasis from recurrent uterine cervix carcinoma presenting as groove pancreatitis. Gastrointest. Endosc..

[8] Ogawa H., Tsujie M., Miyamoto A., Yasui M., Ikenaga M., Hirao M., Fujitani K., Mishima H., Tsujinaka T., Nakamori S. (2011). Isolated pancreatic metastasis from uterine cervical cancer: A case report. Pancreas.

[9] Ogandaga E., Irigo J., Aissa A., Kadiri S., Berhili S., Allaoui M., Al Bouzidi A., El Majjoui S., Elkacemi H., Kebdani T. (2017). Métastase pancréatique d’un carcinome épidermoïde du col de l’utérus: À propos d’un cas. J. Afr. d’Hepato-Gastroenterol..

[10] Mahajan S., Pandit-Taskar N. (2017). Uncommon Metastasis to the Pancreas from Adenocarcinoma of the Cervix Detected on Surveillance 18F-FDG PET/CT Imaging. Clin. Nucl. Med..

[11] Kopke Túlio M.A.C.B., Horta M.S.F., Bispo M.C.S., Bana Costa T.S.N.E., Chagas C.M.D.B.R. (2018). Pancreatic Metastases as the Initial Manifestation of a Neuroendocrine Carcinoma of the Uterine Cervix. Pancreas.

[12] Kim D.J., Park J.M., Kim J.H., Nam K., Kang C.D., Lee S.J., Lee K., Jeon Y.H. (2019). Pancreatic Metastasis from Adenocarcinoma of the Uterine Cervix. Korean J. Gastroenterol..

[13] Gupta P.K., Lal P., Tiwari A. (2019). A case report of carcinoma of uterine cervix throwing heterochronous metastasis to the skin, spleen, and pancreas: The role of multimodality treatment approach. J. Egypt Natl. Canc. Inst..

[14] Lee E.J., Hwang J., Kim D.W. (2019). Small-cell neuroendocrine carcinoma of the uterine cervix with pancreatic metastasis: A case report and a review of the literature. J. Obstet. Gynaecol..

[15] Kudaravalli P., Adu-Gyamfi K.O., Shahsavari D., Kavuri S., Yap J.E.L., Chandrasekar V. (2022). S1910 A Rare Metastatic Lesion in the Pancreas Presenting With Biliary Obstruction. Am. J. Gastroenterol..

[16] Datta D., Aggarwal D., Balakrishnan S., Varshney V.K., Kumar R. (2023). Metastasis from Cervical Cancer Presenting as a Pancreatic Head Mass—An Unexpected Diagnosis!. J. Gastrointest. Cancer.

[17] Rampersad A., Martin E., Valente K., Jones W., Bolton W., Oza V. (2022). S2882 Uterine Cervical Squamous Cell Carcinoma: A Rare Cause of Biliary Obstruction. Am. J. Gastroenterol..

[18] Ye H., Yi X., Li X., Wang J., Zhang J., Liu Z., Lu Z. (2022). Obstructive jaundice due to pancreatic metastasis from cervical squamous cell carcinoma: A case report. J. Clin. Hepatol..

[19] Nakajima Y., Iwasaki E., Kayashima A., Machida Y., Kawasaki S., Horibe M., Kawaida M., Masugi Y., Iwata T., Kanai T. (2023). Successful radiotherapy for recurrent obstructive pancreatitis secondary to pancreatic metastasis from cervical squamous-cell carcinoma. Clin. J. Gastroenterol..

[20] Liao H., Chang X., Hou X., Zhang F. (2025). Pancreatic metastasis from cervical cancer: A case report and literature review. Oncol. Lett..

[21] Marth C., Landoni F., Mahner S., McCormack M., Gonzalez-Martin A., Colombo N. (2017). Cervical cancer: ESMO Clinical Practice Guidelines for diagnosis, treatment and follow-up. Ann. Oncol..

[22] Tewari K.S. (2025). Cervical Cancer. N. Engl. J. Med..

[23] Gardner A.B., Charo L.M., Mann A.K., Kapp D.S., Eskander R.N., Chan J.K. (2020). Ovarian, uterine, and cervical cancer patients with distant metastases at diagnosis: Most common locations and outcomes. Clin. Exp. Metastasis.

[24] Cibula D., Fischerova D., Potter R., Planchamp F., Querleu D., Avall-Lundqvist E., Meder C.H., Kohler C., Landoni F., Lax S. (2018). The European Society of Gynaecological Oncology/European Society for Radiotherapy and Oncology/European Society of Pathology Guidelines for the Management of Patients With Cervical Cancer. Int. J. Gynecol. Cancer.

[25] Song S.W., Cheng J.F., Liu N., Zhao T.H. (2014). Diagnosis and treatment of pancreatic metastases in 22 patients: A retrospective study. World J. Surg. Oncol..

[26] Akashi Y., Saiura A., Kishi Y., Koga R., Morimura R., Yoshioka R., Yamamoto J., Yamaguchi T. (2010). Outcome after surgical resection of isolated metastases to the pancreas. Hepatogastroenterology.

[27] Tsitouridis I., Diamantopoulou A., Michaelides M., Arvanity M., Papaioannou S. (2010). Pancreatic metastases: CT and MRI findings. Diagn. Interv. Radiol..

[28] Ardengh J.C., Lopes C.V., Kemp R., Venco F., de Lima-Filho E.R., dos Santos J.S. (2013). Accuracy of endoscopic ultrasound-guided fine-needle aspiration in the suspicion of pancreatic metastases. BMC Gastroenterol..

[29] DeWitt J., Jowell P., LeBlanc J., McHenry L., McGreevy K., Cramer H., Volmar K., Sherman S., Gress F. (2005). EUS-guided FNA of pancreatic metastases: A multicenter experience. Gastrointest. Endosc..

[30] Katabathina V.S., Ghannam S., Chen M., Desalme B., Gabos R., Emejulu I., Sandhu P.K., Valente P., Dasyam A.K., Prasad S.R. (2024). Update on Pathologic Conditions, Imaging Findings, Prevention, and Management of Human Papillomavirus–related Neoplasms. Radiographics.

[31] Casajoana A., Fabregat J., Peláez N., Busquets J., Valls C., Leiva D., Secanella L., Lladó L., Ramos E. (2012). Indicaciones y resultados de la resección de metástasis pancreáticas. Experiencia en el Hospital Universitario de Bellvitge. Cir. Esp..

[32] Abu-Rustum N.R., Yashar C.M., Arend R., Barber E., Bradley K., Brooks R., Campos S.M., Chino J., Chon H.S., Crispens M.A. (2023). NCCN Guidelines^®^ Insights Cervical Cancer, Version 1.2024. J. Natl. Compr. Cancer Netw..

[33] Reddy S., Wolfgang C.L. (2009). The role of surgery in the management of isolated metastases to the pancreas. Lancet Oncol..

[34] Jarufe N., McMaster P., Mayer A., Mirza D., Buckels J., Orug T., Tekin K., Bramhall S. (2005). Surgical treatment of metastases to the pancreas. Surgeon.

[35] Stoop T.F., Javed A., Oba A., Koerkamp B.G., Seufferlein T., Wilmink J.W., Besselink M.G. (2025). Pancreatic cancer. Lancet.

[36] Qian S., Villarejo-Campos P., García-Olmo D. (2021). The Role of CAR-T Cells in Peritoneal Carcinomatosis from Gastric Cancer: Rationale, Experimental Work, and Clinical Applications. J. Clin. Med..

[37] Kutle I., Polten R., Stalp J.L., Hachenberg J., Todzey F., Hass R., Zimmermann K., von der Ohe J., von Kaisenberg C., Neubert L. (2024). Anti-Mesothelin CAR-NK cells as a novel targeted therapy against cervical cancer. Front. Immunol..

[38] Schepisi G., Casadei C., Toma I., Poti G., Iaia M.L., Farolfi A., Conteduca V., Lolli C., Ravaglia G., Brighi N. (2021). Immunotherapy and its development for gynecological (Ovarian, endometrial and cervical) tumors: From immune checkpoint inhibitors to chimeric antigen receptor (CAR)-T cell therapy. Cancers.

[39] Domínguez-Prieto V., Qian S., Villarejo-Campos P., Meliga C., González-Soares S., Castellano I.G., Jiménez-Galanes S., García-Arranz M., Guadalajara H., García-Olmo D. (2023). Understanding CAR T cell therapy and its role in ovarian cancer and peritoneal carcinomatosis from ovarian cancer. Front. Oncol..

[40] Yu L., Lanqing G., Huang Z., Xin X., Minglin L., Fa-Hui L., Zou H., Min J. (2023). T cell immunotherapy for cervical cancer: Challenges and opportunities. Front. Immunol..

[41] Ronsini C., Solazzo M.C., Bizzarri N., Ambrosio D., La Verde M., Torella M., Carotenuto R.M., Cobellis L., Colacurci N., De Franciscis P. (2022). Fertility-Sparing Treatment for Early-Stage Cervical Cancer ≥ 2 cm: A Problem with a Thousand Nuances—A Systematic Review of Oncological Outcomes. Ann. Surg. Oncol..

[42] Gwacham N.I., McKenzie N.D., Fitzgerald E.R., Ahmad S., Holloway R.W. (2021). Neoadjuvant chemotherapy followed by fertility sparing surgery in cervical cancers size 2–4 cm; emerging data and future perspectives. Gynecol. Oncol..

