# Supplementary material for: Pancreatic Metastases from Cervical Squamous Cell Carcinoma: Systematic Review of the Literature and Case Report
Source: Biomedicines. 2025 Nov 5;13(11):2713. doi: 10.3390/biomedicines13112713 (PMC12650518; doi:10.3390/biomedicines13112713)
Supplement: Supplementary file 1 [file biomedicines-13-02713-s001.zip › biomedicines-3891008-SM.pdf]

JBI CRITICAL APPRAISAL CHECKLIST FOR CASE REPORTS

Reviewer Romero Triana Diego Alejandro Date July 01/2025

Author Hoon lee Et al. Year 2011 Record Number 1

	Yes	No	Unclear	Not applicable
1. Were patient's demographic characteristics clearly described?	☒	☐	☐	☐
2. Was the patient's history clearly described and presented as a timeline?	☒	☐	☐	☐
3. Was the current clinical condition of the patient on presentation clearly described?	☒	☐	☐	☐
4. Were diagnostic tests or assessment methods and the results clearly described?	☒	☐	☐	☐
5. Was the intervention(s) or treatment procedure(s) clearly described?	☐	☐	☒	☐
6. Was the post-intervention clinical condition clearly described?	☐	☒	☐	☐
7. Were adverse events (harms) or unanticipated events identified and described?	☐	☒	☐	☐
8. Does the case report provide takeaway lessons?	☒	☐	☐	☐

Overall appraisal: Include ☒ Exclude ☐ Seek further info ☐

Comments (Including reason for exclusion)

___

___

___

JBI CRITICAL APPRAISAL CHECKLIST FOR CASE REPORTS

Reviewer Romero Triana Diego Alejandro Date July 01/2025

Author Ogawa Et al. Year 2011 Record Number 2

	Yes	No	Unclear	Not applicable
1. Were patient's demographic characteristics clearly described?	☒	☐	☐	☐
2. Was the patient's history clearly described and presented as a timeline?	☒	☐	☐	☐
3. Was the current clinical condition of the patient on presentation clearly described?	☒	☐	☐	☐
4. Were diagnostic tests or assessment methods and the results clearly described?	☒	☐	☐	☐
5. Was the intervention(s) or treatment procedure(s) clearly described?	☒	☐	☐	☐
6. Was the post-intervention clinical condition clearly described?	☒	☐	☐	☐
7. Were adverse events (harms) or unanticipated events identified and described?	☐	☒	☐	☐
8. Does the case report provide takeaway lessons?	☒	☐	☐	☐

Overall appraisal: Include ☒ Exclude ☐ Seek further info ☐

Comments (Including reason for exclusion)

____

____

____

JBI CRITICAL APPRAISAL CHECKLIST FOR CASE REPORTS

Reviewer Romero Triana Diego Alejandro Date July 01/2025

Author Ogandaga Et al. Year 2017 Record Number 3

	Yes	No	Unclear	Not applicable
1. Were patient's demographic characteristics clearly described?	☒	☐	☐	☐
2. Was the patient's history clearly described and presented as a timeline?	☒	☐	☐	☐
3. Was the current clinical condition of the patient on presentation clearly described?	☒	☐	☐	☐
4. Were diagnostic tests or assessment methods and the results clearly described?	☒	☐	☐	☐
5. Was the intervention(s) or treatment procedure(s) clearly described?	☐	☒	☐	☐
6. Was the post-intervention clinical condition clearly described?	☒	☐	☐	☐
7. Were adverse events (harms) or unanticipated events identified and described?	☐	☒	☐	☐
8. Does the case report provide takeaway lessons?	☒	☐	☐	☐

Overall appraisal: Include ☒ Exclude ☐ Seek further info ☐

Comments (Including reason for exclusion)

___

___

___

JBI CRITICAL APPRAISAL CHECKLIST FOR CASE REPORTS

Reviewer: Romero Triana Diego Alejandro Date: July 01/2025

Author: Mahajan Et al. Year: 2017 Record Number: 4

	Yes	No	Unclear	Not applicable
1. Were patient's demographic characteristics clearly described?	☐	☒	☐	☐
2. Was the patient's history clearly described and presented as a timeline?	☒	☐	☐	☐
3. Was the current clinical condition of the patient on presentation clearly described?	☒	☐	☐	☐
4. Were diagnostic tests or assessment methods and the results clearly described?	☒	☐	☐	☐
5. Was the intervention(s) or treatment procedure(s) clearly described?	☒	☐	☐	☐
6. Was the post-intervention clinical condition clearly described?	☐	☒	☐	☐
7. Were adverse events (harms) or unanticipated events identified and described?	☐	☒	☐	☐
8. Does the case report provide takeaway lessons?	☒	☐	☐	☐

Overall appraisal: Include ☒ Exclude ☐ Seek further info ☐

Comments (Including reason for exclusion)

____

JBI CRITICAL APPRAISAL CHECKLIST FOR CASE REPORTS

Reviewer Romero Triana Diego Alejandro Date July 01/2025

Author Kim Et al. Year 2018 Record Number 5

	Yes	No	Unclear	Not applicable
1. Were patient's demographic characteristics clearly described?	☒	☐	☐	☐
2. Was the patient's history clearly described and presented as a timeline?	☒	☐	☐	☐
3. Was the current clinical condition of the patient on presentation clearly described?	☒	☐	☐	☐
4. Were diagnostic tests or assessment methods and the results clearly described?	☒	☐	☐	☐
5. Was the intervention(s) or treatment procedure(s) clearly described?	☐	☒	☐	☐
6. Was the post-intervention clinical condition clearly described?	☐	☒	☐	☐
7. Were adverse events (harms) or unanticipated events identified and described?	☐	☒	☐	☐
8. Does the case report provide takeaway lessons?	☒	☐	☐	☐

Overall appraisal: Include ☒ Exclude ☐ Seek further info ☐

Comments (Including reason for exclusion)

____

____

____

JBI CRITICAL APPRAISAL CHECKLIST FOR CASE REPORTS

Reviewer Romero Triana Diego Alejandro Date July 01/2025

Author Kopke Et al. Year 2018 Record Number 6

	Yes	No	Unclear	Not applicable
1. Were patient's demographic characteristics clearly described?	☒	☐	☐	☐
2. Was the patient's history clearly described and presented as a timeline?	☒	☐	☐	☐
3. Was the current clinical condition of the patient on presentation clearly described?	☒	☐	☐	☐
4. Were diagnostic tests or assessment methods and the results clearly described?	☒	☐	☐	☐
5. Was the intervention(s) or treatment procedure(s) clearly described?	☒	☐	☐	☐
6. Was the post-intervention clinical condition clearly described?	☒	☐	☐	☐
7. Were adverse events (harms) or unanticipated events identified and described?	☐	☒	☐	☐
8. Does the case report provide takeaway lessons?	☒	☐	☐	☐

Overall appraisal: Include ☒ Exclude ☐ Seek further info ☐

Comments (Including reason for exclusion)

____

____

____

JBI CRITICAL APPRAISAL CHECKLIST FOR CASE REPORTS

Reviewer Romero Triana Diego Alejandro Date July 01/2025

Author Gupta Et al. Year 2019 Record Number 7

	Yes	No	Unclear	Not applicable
1. Were patient's demographic characteristics clearly described?	☒	☐	☐	☐
2. Was the patient's history clearly described and presented as a timeline?	☒	☐	☐	☐
3. Was the current clinical condition of the patient on presentation clearly described?	☒	☐	☐	☐
4. Were diagnostic tests or assessment methods and the results clearly described?	☒	☐	☐	☐
5. Was the intervention(s) or treatment procedure(s) clearly described?	☒	☐	☐	☐
6. Was the post-intervention clinical condition clearly described?	☐	☒	☐	☐
7. Were adverse events (harms) or unanticipated events identified and described?	☐	☒	☐	☐
8. Does the case report provide takeaway lessons?	☒	☐	☐	☐

Overall appraisal: Include ☒ Exclude ☐ Seek further info ☐

Comments (Including reason for exclusion)

____

____

____

JBI CRITICAL APPRAISAL CHECKLIST FOR CASE REPORTS

Reviewer Romero Triana Diego Alejandro Date July 01/2025

Author Lee Et al Year 2019 Record Number 8

	Yes	No	Unclear	Not applicable
1. Were patient's demographic characteristics clearly described?	☒	☐	☐	☐
2. Was the patient's history clearly described and presented as a timeline?	☒	☐	☐	☐
3. Was the current clinical condition of the patient on presentation clearly described?	☒	☐	☐	☐
4. Were diagnostic tests or assessment methods and the results clearly described?	☒	☐	☐	☐
5. Was the intervention(s) or treatment procedure(s) clearly described?	☒	☐	☐	☐
6. Was the post-intervention clinical condition clearly described?	☐	☒	☐	☐
7. Were adverse events (harms) or unanticipated events identified and described?	☐	☒	☐	☐
8. Does the case report provide takeaway lessons?	☒	☐	☐	☐

Overall appraisal: Include ☒ Exclude ☐ Seek further info ☐

Comments (Including reason for exclusion)

____

JBI CRITICAL APPRAISAL CHECKLIST FOR CASE REPORTS

Reviewer: Romero Triana Diego Alejandro Date: July 01/2025

Author: Kudarcavalle Et al. Year: 2022 Record Number: 9

	Yes	No	Unclear	Not applicable
1. Were patient's demographic characteristics clearly described?	☒	☐	☐	☐
2. Was the patient's history clearly described and presented as a timeline?	☒	☐	☐	☐
3. Was the current clinical condition of the patient on presentation clearly described?	☒	☐	☐	☐
4. Were diagnostic tests or assessment methods and the results clearly described?	☒	☐	☐	☐
5. Was the intervention(s) or treatment procedure(s) clearly described?	☒	☐	☐	☐
6. Was the post-intervention clinical condition clearly described?	☐	☒	☐	☐
7. Were adverse events (harms) or unanticipated events identified and described?	☐	☒	☐	☐
8. Does the case report provide takeaway lessons?	☒	☐	☐	☐

Overall appraisal: Include ☒ Exclude ☐ Seek further info ☐

Comments (Including reason for exclusion)

____

____

____

JBI CRITICAL APPRAISAL CHECKLIST FOR CASE REPORTS

Reviewer Romero Triana Diego Alejandro Date July 01/2025

Author Datta Et al. Year 2022 Record Number 10

	Yes	No	Unclear	Not applicable
1. Were patient's demographic characteristics clearly described?	☒	☐	☐	☐
2. Was the patient's history clearly described and presented as a timeline?	☒	☐	☐	☐
3. Was the current clinical condition of the patient on presentation clearly described?	☒	☐	☐	☐
4. Were diagnostic tests or assessment methods and the results clearly described?	☒	☐	☐	☐
5. Was the intervention(s) or treatment procedure(s) clearly described?	☒	☐	☐	☐
6. Was the post-intervention clinical condition clearly described?	☒	☐	☐	☐
7. Were adverse events (harms) or unanticipated events identified and described?	☐	☒	☐	☐
8. Does the case report provide takeaway lessons?	☒	☐	☐	☐

Overall appraisal: Include ☒ Exclude ☐ Seek further info ☐

Comments (Including reason for exclusion)

____

____

____

JBI CRITICAL APPRAISAL CHECKLIST FOR CASE REPORTS

Reviewer Romero Triana Diego Alejandro Date July 01/2025

Author Rampersad Et al. Year 2022 Record Number 11

	Yes	No	Unclear	Not applicable
1. Were patient's demographic characteristics clearly described?	☒	☐	☐	☐
2. Was the patient's history clearly described and presented as a timeline?	☒	☐	☐	☐
3. Was the current clinical condition of the patient on presentation clearly described?	☒	☐	☐	☐
4. Were diagnostic tests or assessment methods and the results clearly described?	☒	☐	☐	☐
5. Was the intervention(s) or treatment procedure(s) clearly described?	☐	☒	☐	☐
6. Was the post-intervention clinical condition clearly described?	☐	☒	☐	☐
7. Were adverse events (harms) or unanticipated events identified and described?	☐	☒	☐	☐
8. Does the case report provide takeaway lessons?	☒	☐	☐	☐

Overall appraisal: Include ☒ Exclude ☐ Seek further info ☐

Comments (Including reason for exclusion)

____

____

____

JBI CRITICAL APPRAISAL CHECKLIST FOR CASE REPORTS

Reviewer Romero Triana Diego Alejandro Date July 01/2025

Author YE Hao Et al. Year 2022 Record Number 12

	Yes	No	Unclear	Not applicable
1. Were patient's demographic characteristics clearly described?	☒	☐	☐	☐
2. Was the patient's history clearly described and presented as a timeline?	☒	☐	☐	☐
3. Was the current clinical condition of the patient on presentation clearly described?	☒	☐	☐	☐
4. Were diagnostic tests or assessment methods and the results clearly described?	☒	☐	☐	☐
5. Was the intervention(s) or treatment procedure(s) clearly described?	☒	☐	☐	☐
6. Was the post-intervention clinical condition clearly described?	☒	☐	☐	☐
7. Were adverse events (harms) or unanticipated events identified and described?	☐	☒	☐	☐
8. Does the case report provide takeaway lessons?	☒	☐	☐	☐

Overall appraisal: Include ☒ Exclude ☐ Seek further info ☐

Comments (Including reason for exclusion)

___

___

___

JBI CRITICAL APPRAISAL CHECKLIST FOR CASE REPORTS

Reviewer Romero Triana Diego Alejandro Date July 01/2025

Author Nakajima Et al Year 2023 Record Number 13

	Yes	No	Unclear	Not applicable
1. Were patient's demographic characteristics clearly described?	☒	☐	☐	☐
2. Was the patient's history clearly described and presented as a timeline?	☒	☐	☐	☐
3. Was the current clinical condition of the patient on presentation clearly described?	☒	☐	☐	☐
4. Were diagnostic tests or assessment methods and the results clearly described?	☒	☐	☐	☐
5. Was the intervention(s) or treatment procedure(s) clearly described?	☒	☐	☐	☐
6. Was the post-intervention clinical condition clearly described?	☒	☐	☐	☐
7. Were adverse events (harms) or unanticipated events identified and described?	☐	☒	☐	☐
8. Does the case report provide takeaway lessons?	☒	☐	☐	☐

Overall appraisal: Include ☒ Exclude ☐ Seek further info ☐

Comments (Including reason for exclusion)

____

____

____

JBI CRITICAL APPRAISAL CHECKLIST FOR CASE REPORTS

Reviewer Romero Triana Diego Alejandro Date July 01/2025

Author Liao Et al. Year 2024 Record Number 14

	Yes	No	Unclear	Not applicable
1. Were patient's demographic characteristics clearly described?	☒	☐	☐	☐
2. Was the patient's history clearly described and presented as a timeline?	☒	☐	☐	☐
3. Was the current clinical condition of the patient on presentation clearly described?	☒	☐	☐	☐
4. Were diagnostic tests or assessment methods and the results clearly described?	☒	☐	☐	☐
5. Was the intervention(s) or treatment procedure(s) clearly described?	☒	☐	☐	☐
6. Was the post-intervention clinical condition clearly described?	☒	☐	☐	☐
7. Were adverse events (harms) or unanticipated events identified and described?	☐	☒	☐	☐
8. Does the case report provide takeaway lessons?	☒	☐	☐	☐

Overall appraisal: Include ☒ Exclude ☐ Seek further info ☐

Comments (Including reason for exclusion)

____

____

____

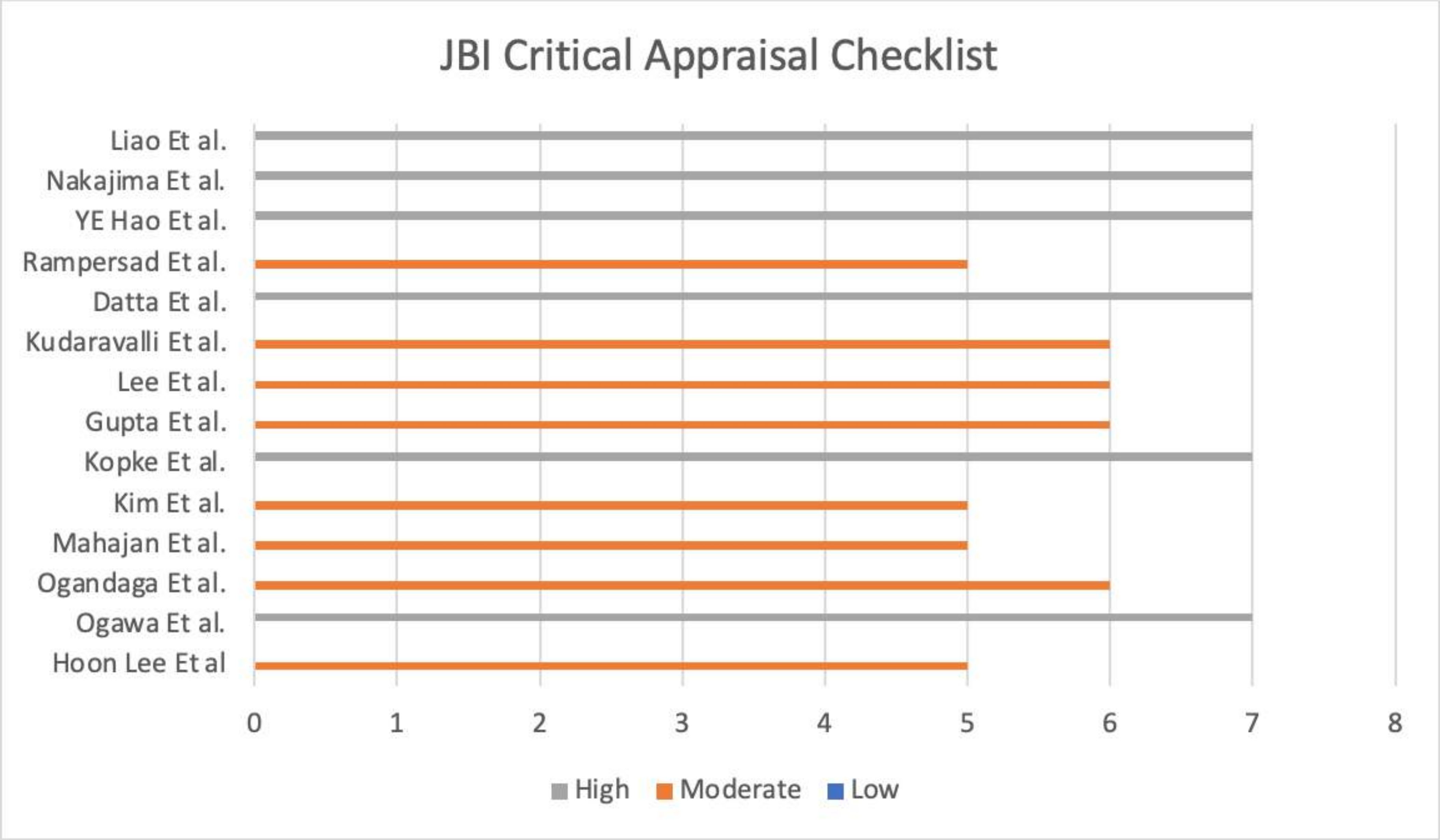

JBI Critical Appraisal Checklist
Liao Et al.
Nakajima Et al.
YE Hao Et al.
Rampersad Et al.
Datta Et al.
Kudaravalli Et al.
Lee Et al.
Gupta Et al.
Kopke Et al.
Kim Et al.
Mahajan Et al.
Ogandaga Et al.
Ogawa Et al.
Hoon Lee Et al
0
1
2
3
4
5
6
7
8
High
Moderate
Low

PRISMA

PRISMA 2020 Checklist

Section and Topic	Item #	Checklist item	Location where item is reported
**TITLE**			
Title	1	Identify the report as a systematic review.	Title: *"Pancreatic Metastases from Cervical Squamous Cell Carcinoma: Systematic Review of the Literature and Case Report"*
**ABSTRACT**			
Abstract	2	See the PRISMA 2020 for Abstracts checklist.	Structured abstract at the beginning of the manuscript
**INTRODUCTION**			
Rationale	3	Describe the rationale for the review in the context of existing knowledge.	Introduction:<br>Paragraph 1, 2, 3
Objectives	4	Provide an explicit statement of the objective(s) or question(s) the review addresses.	Introduction:<br>Paragraph 4
**METHODS**			
Eligibility criteria	5	Specify the inclusion and exclusion criteria for the review and how studies were grouped for the syntheses.	Material and methods: "Inclusion and exclusion criteria"
Information sources	6	Specify all databases, registers, websites, organisations, reference lists and other sources searched or consulted to identify studies. Specify the date when each source was last searched or consulted.	Material and methods: "Systematic review design and methodological quality assessment"
Search strategy	7	Present the full search strategies for all databases, registers and websites, including any filters and limits used.	Material and methods: "Systematic review design and methodological quality assessment"
Selection process	8	Specify the methods used to decide whether a study met the inclusion criteria of the review, including how many reviewers screened each record and each report retrieved, whether they worked independently, and if applicable, details of automation tools used in the process.	Material and methods: "Systematic review design and methodological quality assessment"
Data collection process	9	Specify the methods used to collect data from reports, including how many reviewers collected data from each report, whether they worked independently, any processes for obtaining or confirming data from study investigators, and if applicable, details of automation tools used in the process.	Material and methods: "Systematic review design and methodological quality assessment"
Data items	10a	List and define all outcomes for which data were sought. Specify whether all results that were compatible with each outcome domain in each study were sought (e.g. for all measures, time points, analyses), and if not, the methods used to decide which results to collect.	Material and methods: "Table 1"<br>Results: "Search Results

Section and Topic	Item #	Checklist item	Location where item is reported
			and Study Selection and study characteristics”
	10b	List and define all other variables for which data were sought (e.g. participant and intervention characteristics, funding sources). Describe any assumptions made about any missing or unclear information.	Material and methods: “Table 1”<br>Results: “Search Results and Study Selection and study characteristics”
Study risk of bias assessment	11	Specify the methods used to assess risk of bias in the included studies, including details of the tool(s) used, how many reviewers assessed each study and whether they worked independently, and if applicable, details of automation tools used in the process.	Material and methods: “Systematic review design and methodological quality assessment”
Effect measures	12	Specify for each outcome the effect measure(s) (e.g. risk ratio, mean difference) used in the synthesis or presentation of results.	Material and methods: “Systematic review design and methodological quality assessment”
Synthesis methods	13a	Describe the processes used to decide which studies were eligible for each synthesis (e.g. tabulating the study intervention characteristics and comparing against the planned groups for each synthesis (item #5)).	Material and methods: “Systematic review design and methodological quality assessment” and “table 1”<br>Results: “Search Results and Study Selection and study characteristics”
	13b	Describe any methods required to prepare the data for presentation or synthesis, such as handling of missing summary statistics, or data conversions.	Material and methods: “Systematic review design and methodological quality assessment”<br>Results: “Search Results and Study Selection and study characteristics”<br>(Only narrative synthesis, no meta-analisis)
	13c	Describe any methods used to tabulate or visually display results of individual studies and syntheses.	Material and methods: “Systematic review design and methodological quality assessment”<br>Results: “Search Results and Study Selection and study characteristics”<br>(Only narrative synthesis, no meta-analisis)

Section and Topic	Item #	Checklist item	Location where item is reported
	13d	Describe any methods used to synthesize results and provide a rationale for the choice(s). If meta-analysis was performed, describe the model(s), method(s) to identify the presence and extent of statistical heterogeneity, and software package(s) used.	Material and methods: "Systematic review design and methodological quality assessment"<br>Results: "Search Results and Study Selection and study characteristics"<br><br>(Only narrative synthesis, no meta-analisis)
	13e	Describe any methods used to explore possible causes of heterogeneity among study results (e.g. subgroup analysis, meta-regression).	Material and methods: "Systematic review design and methodological quality assessment"<br>Results: "Search Results and Study Selection and study characteristics"<br><br>(Only narrative synthesis, no meta-analisis)
	13f	Describe any sensitivity analyses conducted to assess robustness of the synthesized results.	Material and methods: "Systematic review design and methodological quality assessment"<br>Results: "Search Results and Study Selection and study characteristics"<br><br>(Only narrative synthesis, no meta-analisis)
Reporting bias assessment	14	Describe any methods used to assess risk of bias due to missing results in a synthesis (arising from reporting biases).	Material and methods: "Systematic review design and methodological quality assessment"<br>Results: "Search Results and Study Selection and study characteristics"
Certainty assessment	15	Describe any methods used to assess certainty (or confidence) in the body of evidence for an outcome.	Material and methods: "Systematic review design and methodological quality assessment"

PRISMA 2020 Checklist

Section and Topic	Item #	Checklist item	Location where item is reported
**RESULTS**			
Study selection	16a	Describe the results of the search and selection process, from the number of records identified in the search to the number of studies included in the review, ideally using a flow diagram.	Material and methods: "Figure 2"<br>Results: "Search Results and Study Selection and study characteristics
	16b	Cite studies that might appear to meet the inclusion criteria, but which were excluded, and explain why they were excluded.	Material and methods: "Figure 2"<br>Results: "Search Results and Study Selection and study characteristics
Study characteristics	17	Cite each included study and present its characteristics.	Material and methods: "Table 1"
Risk of bias in studies	18	Present assessments of risk of bias for each included study.	Material and methods: "Systematic review design and methodological quality assessment"
Results of individual studies	19	For all outcomes, present, for each study: (a) summary statistics for each group (where appropriate) and (b) an effect estimate and its precision (e.g. confidence/credible interval), ideally using structured tables or plots.	Material and methods: "Table 1"<br>(Only narrative)
Results of syntheses	20a	For each synthesis, briefly summarise the characteristics and risk of bias among contributing studies.	Results<br>(Only narrative, no meta-analisis)
	20b	Present results of all statistical syntheses conducted. If meta-analysis was done, present for each the summary estimate and its precision (e.g. confidence/credible interval) and measures of statistical heterogeneity. If comparing groups, describe the direction of the effect.	Results<br>(Only narrative, no meta-analisis)
	20c	Present results of all investigations of possible causes of heterogeneity among study results.	Results<br>(Only narrative, no meta-analisis)
	20d	Present results of all sensitivity analyses conducted to assess the robustness of the synthesized results.	Results<br>(Only narrative, no meta-analisis)
Reporting biases	21	Present assessments of risk of bias due to missing results (arising from reporting biases) for each synthesis assessed.	Results

PRISMA

PRISMA 2020 Checklist

Section and Topic	Item #	Checklist item	Location where item is reported
			Discussion<br><br>(Only narrative, no meta-analisis)
Certainty of evidence	22	Present assessments of certainty (or confidence) in the body of evidence for each outcome assessed.	Results<br>Discussion<br><br>(Only narrative, no meta-analisis)
**DISCUSSION**			
Discussion	23a	Provide a general interpretation of the results in the context of other evidence.	Discussion:<br>Paragraph 2-12, 15.
	23b	Discuss any limitations of the evidence included in the review.	Discussion:<br>Paragraph 1, 10, 13,
	23c	Discuss any limitations of the review processes used.	Discussion:<br>Last paragraph
	23d	Discuss implications of the results for practice, policy, and future research.	Discussion:<br>Last paragraph
**OTHER INFORMATION**			
Registration and protocol	24a	Provide registration information for the review, including register name and registration number, or state that the review was not registered.	Material and methods: “Systematic review design and methodological quality assessment”
	24b	Indicate where the review protocol can be accessed, or state that a protocol was not prepared.	Material and methods: “Systematic review design and methodological quality assessment”
	24c	Describe and explain any amendments to information provided at registration or in the protocol.	Material and methods: “Systematic review design and methodological quality assessment”
Support	25	Describe sources of financial or non-financial support for the review, and the role of the funders or sponsors in the review.	Funding
Competing interests	26	Declare any competing interests of review authors.	Conflicts of Interest
Availability of data, code and other materials	27	Report which of the following are publicly available and where they can be found: template data collection forms; data extracted from included studies; data used for all analyses; analytic code; any other materials used in the review.	Data Availability Statement

PRISMA

PRISMA 2020 Checklist

From: Page MJ, McKenzie JE, Bossuyt PM, Boutron I, Hoffmann TC, Mulrow CD, et al. The PRISMA 2020 statement: an updated guideline for reporting systematic reviews. BMJ 2021;372:n71. doi: 10.1136/bmj.n71.
This work is licensed under CC BY 4.0. To view a copy of this license, visit https://creativecommons.org/licenses/by/4.0/

Database	Controlled vocabulary	Search string	Search period
Pubmed	MeSH: “Pancreatic Neoplasms”, “Uterine Cervical Neoplasm”, “Neoplasm Metastasis”, “Pancreas”	(“("Pancreatic Neoplasms"[MeSH]) AND ("Uterine Cervical Neoplasms"[MeSH]) OR (("Uterine Cervical Neoplasms"[MeSH]) AND ("Neoplasm Metastasis"[MeSH]) AND ("Pancreas"[MeSH]))	March – April 2025 October 20205
Embase	Emtree: ‘pancreas tumor’, ‘uterine cervix cancer’, ‘pancreas metastasis’	(('pancreatic metastasis'/exp OR 'pancreas metastasis':ti,ab OR 'pancreatic tumor':ti,ab) AND ('cervical cancer'/exp OR 'uterine cervix cancer':ti,ab OR 'uterine cervical neoplasm':ti,ab))	March – April 2025 October 20205
Cochrane Library	Keywords: “pancreatic mass”, “cervical cancer”, “pancreatic metastases”	("pancreatic neoplasm*" OR "pancreatic mass*" OR "pancreas metastas*") AND ("uterine cervical neoplasm*" OR "cervical cancer" OR "cervix carcinoma")	March – April 2025 October 20205
